# COVID-19 Pandemic: An Insult Over Injury for Lebanon

**DOI:** 10.1007/s10900-020-00884-y

**Published:** 2020-07-13

**Authors:** Abdul Rahman Bizri, Hussein H. Khachfe, Mohamad Y. Fares, Umayya Musharrafieh

**Affiliations:** 1 Division of Infectious Diseases, Department of Internal Medicine, American University of Beirut Medical Center, Beirut, Lebanon; 2 National Committee for Communicable Diseases, Beirut, Lebanon; 3 COVID-19 Taskforce, Lebanese Society for Infectious Diseases and Clinical Microbiology, Beirut, Lebanon; 4 Faculty of Medicine, American University of Beirut Medical Center, P.O. Box: 11–0236, Beirut, Lebanon; 5 Neuroscience Research Center, Faculty of Medical Sciences, Lebanese University, Beirut, Lebanon; 6 College of Medical, Veterinary, and Life Sciences, University of Glasgow, Glasgow, Scotland UK; 7 Department of Family Medicine, Director, COVID-19 Clinic, American University of Beirut Medical Center, Beirut, Lebanon

**Keywords:** Novel coronavirus disease 2019, COVID-19, Lebanon, Infectious Diseases

## Abstract

The outbreak of the novel coronavirus disease in 2019 (COVID-19) caused a plethora of challenges worldwide and tested healthcare systems across the six continents. Lebanon had recently faced harsh political and economic crises. We aim to describe the effect of COVID-19 on an already crisis-stricken country. A descriptive analysis of the burden of COVID-19 pandemic on Lebanon was performed. Relevant data on COVID-19 was retrieved from the Lebanese Ministry of Public Health from February 21 till June 13th, 2020. Results obtained were analyzed and a literature review was performed. 1422 confirmed COVID-19 cases were identified and reported in Lebanon by June 13th, 2020, comprising an incidence rate of 208/million persons. There has been a total of 31 deaths thus far, with a reported death rate of 5/million persons. The age group with the highest number of cases was 20–29 years. Beirut was the district with the highest number of cases (18%). The COVID-19 crisis has impacted the country on a multifactorial level. COVID-19 could not have come at a worse time for Lebanon. The country is on brink of bankruptcy, the healthcare system is struggling for survival and the government is striving to regain the trust of the population.

## Introduction

Regardless of the sizeable progress in the fields of medicine and public health, infectious diseases continue to present a substantial threat to humanity [1]. The impact of infectious diseases on population health has been traditionally measured by the number of cases and deaths attributable to a specific infection. Increasingly, the impact and burden of disease beyond individual health is being considered. In addition, socioeconomic consequences are well-recognized externalities related to the spread of infectious diseases in a population, and accordingly, should be dealt with high regard [2].

The first documented case of the novel coronavirus 2019 (COVID-19) infection in Lebanon was on February 21st, 2020 [3]. Since then, various aspects of life in the country have been seriously or partially disrupted. The Lebanese population, who were heavily indulged in a popular uprising against corruption and political mismanagement, were about to face another serious adversary [4]. On October 17, 2019 the people of Lebanon started an unprecedented rebellion resulting in civil unrest and political, as well as social, turmoil. The immediate impact was the collapse of the banking system, and putting the country on the brink of bankruptcy [5]. The COVID-19 pandemic could not have come at a worse time for the citizens of Lebanon.

To our knowledge, this is the first report from the Middle East about the impact of COVID-19 on the health system, economy, education and the psychological wellbeing of the population.

## Methods

In this descriptive analysis we attempt to assess the burden of the COVID-19 pandemic on the Lebanese population relevant to: (1) the number of cases and attributed mortality, date of infection, district, gender and age distribution, (2) its impact on the healthcare, economic, and educational sectors, and the psychological trauma associated with the fear from infection and stress of quarantine. Deidentified and publicly accessible data from the Lebanese Ministry of Public Health (LMOPH) Epidemiologic Surveillance Unit about COVID-19 confirmed cases up until June 13th were obtained [3]. A relevant literature review was conducted as well.

## Results

As of June 13th, 2020, 1422 cases of COVID-19 have been reported in Lebanon, with a rate of 208/ million persons. Out of these cases, 811 patients (57%) were males, and 611 patients (43%) were females. Around 782 cases (55%) have been considered mild cases, 564 cases (39.7%) have been asymptomatic, and 76 cases (5.3%) have been described as severe. There has been a total of 31 reported deaths thus far, with a subsequent death rate of 5/million persons and 2.1% of those reported.

**Fig. 1:**
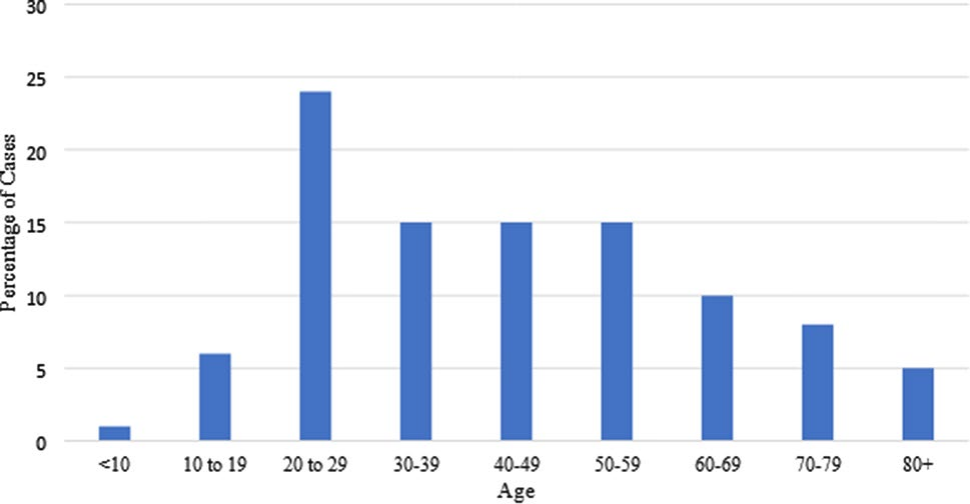
The distribution of COVID-19 cases in Lebanon by age groups

Distribution of COVID-19 cases per age group approximates a normal distribution (Fig. 1). The age group with the highest number of cases was the 20–29 years, with 356 cases (25%), while the age groups with the lowest number were 70–79 years and < 10 years, with 73 cases (5%) and 92 cases (6.44%) respectively (Fig. 1). The districts with the highest number of confirmed cases were the Beirut and the El Maten with 252 cases (18%) and 165 cases (12%) respectively (Table 1).

**Table 1:** Distribution of novel coronavirus disease 2019 (COVID-19) cases across the different Lebanese governorates as of June 13th, 2020

Place of residence	Total number of confirmed cases	Percentage (%)
Akkar	78	5
Aley	54	4
Baabda	121	8
Baalbek	19	1
Batroun	25	2
Bcharre	74	5
Beirut	252	18
Bent Jbeil	12	1
Chouf	131	9
El Maten	165	12
El-Minieh	37	3
Hermel	2	0
Jbeil	56	4
Jezzine	5	0
Kesewen	85	6
Koura	12	1
Marjayoun	8	1
Nabatieh	22	2
Rachaya	3	0
Saida	27	2
Sour	45	3
Tripoli	33	2
West Bekaa	14	1
Zahle	104	7
Zgharta	48	3
Total	1432	100

**Fig. 2:**
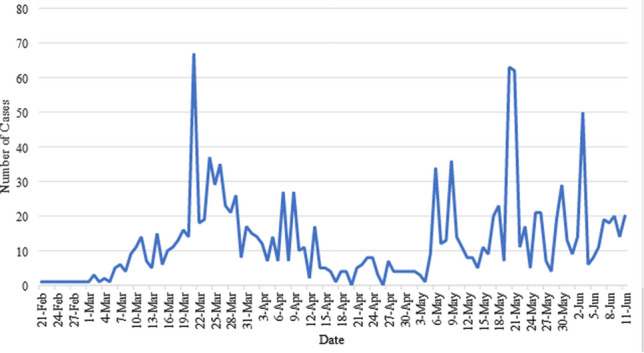
Number of new COVID-19 cases in Lebanon starting 21 February, and up till June 12th, 2020

**Fig. 3:**
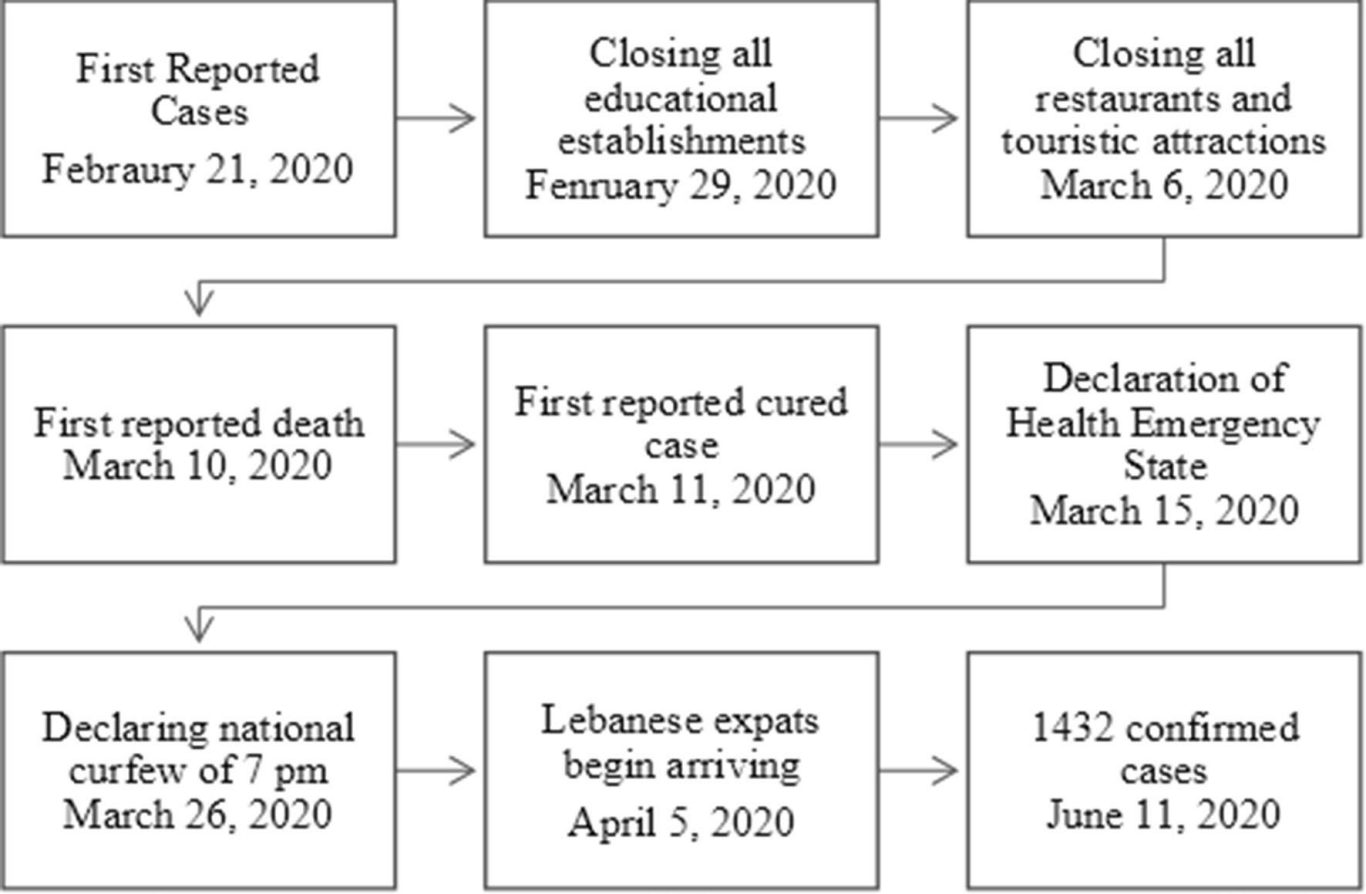
Timeline showing the major COVID-19 events taking place in Lebanon

Of the total number of cases, 768 cases (54%) were attributed to contact with a confirmed case, 441 cases (31%) were attributed to travel-related affairs, 171 cases (12%) were still under investigation, and 28 cases (2%) had unidentified sources of infection. The trend in the number of cases and their incidence can be seen in Fig. 2. March 20th recorded the highest number of new cases with 53 cases/day. Two other spikes occurred at around 22nd of May, and the 1st of June. A timeline of the major events related to the COVID-19 disease in Lebanon can be visualized in Fig. 3.

The impact of COVID-19 further extended beyond morbidity and mortality, and affected the health, socioeconomic and educational sectors of the country, as well as the psychological welfare of the population.

## Discussion

The incidence rate of COVID-19 cases in Lebanon was 208/million persons, with a mortality rate of 5/million persons. This rate is considerably lower than the global average incidence of 989/million persons and average mortality of 54.8/million persons [6].This can be attributed to several demographic and logistic factors. Most of our cases were asymptomatic or mild cases that belonged to the younger population, as the majority of the patients (84%) were younger than 59 years old. This is noticeably younger than the age distribution of other countries who were highly affected by the disease [7, 8]. Many of these patients were workers or students studying abroad and were exposed to the virus in other countries. This explains the fact why 80% of the cases in the country were due to contact with a confirmed case or travel-related affairs. The younger affected population in Lebanon may explain the lower mortality rate present in the Lebanese population. All positively-tested patients, irrespective of the severity, were advised admission as per LMOPH.

In addition, strict measures were undertaken by the Lebanese government, and these included testing all people possibly exposed by travel-related affairs, or showing symptoms as per World Health Organization (WHO) recommendations. Initiating a national curfew helped limit the people’s mobility and the disease’s spread (Fig. 3). And add to that the scarcity of public spaces and public transportation services in Lebanon, one can understand how the spread of the disease in Lebanon was more contained than in other countries [9].

On March 20th, a spike in incidence was noted (Fig. 2). Multiple factors could have contributed to this finding. The WHO and the LMOPH initially approved only one center at Rafik Hariri University Hospital (RHUH) to conduct PCR testing for COVID-19. Later, other medical center labs were included as certified testing centers. The backlog of tests that were not performed due to inability of RHUH to cope with alone, were distributed on all available medical centers, and all the results were reported at that date (Table 1). Other spikes that occurred after April 5 were due to the return of the Lebanese expats, which caused surges in COVID-19 incidence nationwide (Fig. 3).

The inclusion of other diagnostic centers allowed people more accessibility to seek diagnosis. Our study revealed that Beirut and El Maten districts had the highest number of cases when compared to other regions in the nation. This is due to the highly populous nature of these regions, and the subsequent high risk of human-to-human transmission.

That being said, some may speculate that the low incidence rate in Lebanon is attributed to other reasons. The reported number of tests issued in Lebanon is 15145/1 million, and this number falls short when compared to that of other European and North American countries [6]. The testing policy employed by the LMOPH did not solicit unconditional screening in order to preserve the limited medical resources present [3]. It only issued free testing to cases that fit the definitions of the WHO and the Lebanese Society of Infectious Diseases (LSID). This meant that individuals needed to have relevant symptoms (fever, cough, rhinitis, sore throat or shortness of breath) within 14 days from visiting an endemic foreign country [3]. Asymptomatic patients or patients with no travel history were told to quarantine themselves without any testing or confirmation of diagnosis. In addition, tests conducted in the private sector were not reported to the nation’s registry. As such, this could have underestimated the incidence rates in the country and given a false sense of security, as the reported cases may just well be, the tip of the iceberg.

### Impact on Healthcare System

Lebanon is believed to be the country with the highest number of refugees per capita in the world, and the Lebanese primary health care system was already trying to cope with the extra burden of more than one million Syrian refugees [10–13]. The recent political events following the uprising, and the resulting socioeconomic consequences have shifted the load of medical ambulatory care towards the public sector, mainly the network of primary healthcare centers (PHCC) supported by the government and partnering philanthropic non-governmental organizations. Recent data from the LMOPH showed that there was a 6% annual increase in the number of patients’ visits to the PHCC and a 5.7% increase in the total number of patients [3]. The shifting emphasis towards preparing public-sector hospitals to receive COVID-19 patients and diverting governmental funds has rendered the PHCC resources depleted; and this threatens the continuity of some of their basic services [3]. Not to mention, that the fear of acquiring infection, along with the forced curfew, have refrained many individuals from visiting PHCC clinics, even for fully subsidized services. In some countries, telemedicine was used to suffice for face-to-face encounters, but unfortunately, this option is not currently feasible in Lebanon [11].

The global circulation of the COVID-19 virus, and the resulting pandemic, threaten to disrupt routine and planned campaigns of immunization activities, and decrease the demand for vaccinations. This is due to the physical distancing requirements and the community reluctance. Interruption of immunization services, even for short intervals, will lead to increase in the numbers of susceptible individuals and foster the likelihood of outbreak-prone vaccine preventable diseases (VPDs) such as measles [12]. In Lebanon, routine immunization activities, as well as the pre-planned phase II of the measles vaccination campaign, have been severely interrupted. These are mainly centered around the PHCC clinics that have already been impacted by consequences of the pandemic. Vaccination campaigns are mainly directed towards school children and planned community gatherings in rural areas. Given the fact the schools have closed since February 29, 2020, and recommendations for social distancing prevent any form of gathering, it is appropriate to assume that all forms of immunization activities have been disrupted. The inability to continue with routine vaccination and carry on with the phase II of the measles campaign may yield serious health consequences [13].

Hospitals play a crucial role in the response to communicable disease epidemics [14]. Despite experiencing repeated disastrous events and several military internal conflicts and wars, no Lebanese hospital has prepared “Emergency Risk Management Program” plans for emergencies like infectious diseases epidemics [15]. RHUH, the largest public hospital in Lebanon, was assigned to be the main medical center for diagnosing and managing suspected and confirmed COVID-19 cases. Since then, all international and national resources were diverted towards supporting RHUH COVID-19 designated activities. As the numbers of affected or suspected individuals grew, other medical centers were drawn into the fight, and had their own separate COVID-19 care centers established. All elective surgeries were put on hold, and cold medical cases were managed on ambulatory basis. Medical equipment, tools, and machines needed to fight the virus had been scarce with prominently high prices, and this constituted a major limiting factor in the country [15].

### Impact on Economy

Lebanon is no stranger to economic turmoil. In fact, the debt-to-GDP ratio of the country is expected to reach 160% by the end of 2020 [16]. In addition, and since the beginning of the recent uprisings in Lebanon, also known as the October 17 Revolution, over 700 establishments have closed, sending around 25,000 employees into unemployment [15].All this came at a time where shortage in local and foreign currency took place, governmental regulations and private banks restricted money withdrawals, and transfers were prevented. The lack of funds, resources and foreign currency have positioned the country at a critical spot when faced with the COVID-19 pandemic. Due to the abysmal economic situation in Lebanon, the government was unable to set aside a stimulus package to help equip both public and private hospitals with much needed resources to fight the disease [17]. Public owned hospitals were almost totally dependent on WHO, foreign, and local non-governmental aid to import essential diagnostic kits, personal protection equipment (PPE), and other needed supplies and equipment.

The economic problem does not only affect healthcare establishments, but also, the population at large. The Lebanese government had initiated a state of health emergency, in an attempt to halt the transmission of the disease (Fig. 3). By doing so, the government negatively affected the livelihood of millions of people. Around 30% of Lebanon’s youth are currently unemployed, and thousands of people are day-to-day contracted workers [18]. In addition, hundreds of thousands of citizens rely on their own small businesses, that have been forced to close in order to comply with the health emergency state and the national curfew.

Lebanon has failed to reach economic stability since the conclusion of the civil war back in 1990. The recent popular uprising further complicated the socioeconomic situation in the country. To top everything off, COVID-19 has put the Lebanese healthcare system under the threat of oversaturation, like what happened in other heavily impacted countries. The inability of the government to provide the necessary supplies to the healthcare establishments and the quarantined citizens and to support closed businesses are ominous signs for potential failure.

### Impact on Education

One of the major social downsides of the COVID-19 outbreak is its prominent impact on the educational system [19]. The system was already struggling with the political and national unrest, instigated by the October uprising, and the COVID-19 outbreak managed to exacerbate the situation. This insinuates dire consequences in a country like Lebanon, where resources are limited, and curricula are rigidly time-dependent. By adhering to the WHO’s recommendations, and to avoid further disseminating the disease, the government decided to close all learning institutions on February 29th, 2020 until further notice [20].

A movement towards online-learning was corroborated by most educational facilities. The idea was to enable students to continue learning from within their quarantine, in order to proceed with their designated curricula or courses, and not increase the risk of human-to-human transmission. Recorded lectures and online exams were issued, and students were expected to engage and participate in said activities. Nevertheless, scarce resources and infrastructure in Lebanon have proved to be a major limitation in this setting, as the internet connection has long been below par in the country, and the electrical energy rationing rates have only gotten worse, despite the large sums of money invested [21–23]. The quarantine measures enforced by the government caused a surge in internet traffic over the past weeks, which only managed to further slow the internet services across the country.

In addition, the country’s frail economy along with its concurrent banking crisis had caused many families to be poverty-stricken, and as a result, many of which cannot afford proper devices that can accommodate these learning services [20]. As such, many students remained helpless with regards to this educational dilemma in the country.

Considering the prominent instability in the region, many Lebanese had aspired to graduate and complete their learning milestones, in order to travel abroad and start new lives. Graduation ceremonies were halted until further notice, and administrations started weighing in alterative options. In addition, many high school students became oblivious to what their futures hold, as many questions were raised with regards to assessment and graduation requirements. The International Baccalaureate(IB) program has decided to withhold the April and May examinations, and award diplomas on the basis of students’ coursework and established assessments [24]. On the other hand, the Lebanese government cancelled the Lebanese Baccalaureate examinations this summer, leaving many students uneasy and anxious about their future [7, 25]. The COVID-19 outbreak put a dent in the Lebanese educational sector, caused a delay in educational pillars, and left the nation and its students in limbo.

### Impact on Psychological Wellbeing

One of the major steps in the management of the COVID-19 outbreak was isolation and quarantine. This imposes multifactorial psychological burdens, and presents tough challenges to the people’s mental wellbeing. Besides the fear of experiencing any physical symptoms potentially related to the infection, people fear from spreading the infection to others. The loss of usual routines and activities causes significant frustration and unrest. The Lebanese society has often been described as incredibly resilient and sociable [23, 26]. Nevertheless, quarantine causes high levels of social loneliness, as people will be deprived of their ability to take part in their usual routines and social activities.

The duration of quarantine is an important variable to factor in when assessing the impact of COVID-19 on the psychological welfare of the people. Studies show that the longer the quarantine is, the higher the association with poor mental health, post-traumatic stress symptoms, and avoidance [27, 28]. It has been shown that those under quarantine for more than 10 days had higher psychological symptoms than those with lesser periods. In Lebanon, quarantine was imposed across all the population and was expected to last for weeks.

The ability to ensure adequate supplies of essential items during quarantine period is an additional reason for concern [29]. Given the frail situation that resulted from the political crises experienced before the COVID-19 outbreak, the Lebanese population were left to guarantee all their needs on their own, amidst emergence of monopolization and expropriation [30]. This shortage in resources, along with opportunism of some businesses, have led to a state of panic in the Lebanese community.

The Lebanese are also suffering significant financial losses during the quarantine, as many jobs were suspended, and many professional events were cancelled or delayed. These losses have further augmented the tight situation, especially considering the financial deprivation caused by the concurrent economic crisis in Lebanon. At a time when mass quarantine is inevitable to stop the spread of the virus, and the outbreak is as much an existential threat as it is a medical threat, COVID-19 comes as a crisis in a crisis [31]. Already suffering under a moribund economy, the Lebanese were forced to shut down shops, gyms, pubs and theatres to stop the spread of the virus. The bad financial situation constituted a risk factor for many psychological disorders, as many Lebanese worried and feared their inability to provide and care for their families [32].

Lebanese people were flooded with information from different media outlets, many of which were neither credible nor well-sourced. The power of the social media had its bearings; the quick spread of rumors, misinformation, and fake news, increased the fear, and created new challenges to the Lebanese authorities to deal with [33]. This affected many Lebanese who speculated a lack of transparency from health officials regarding the severity of the pandemic.

Finally, one of the most important psychological burdens suffered by infected or suspected COVID-19 patients was the stigma of having the disease. This includes patients, those exposed, and more significantly, healthcare workers. Those quarantined were most likely to be stigmatized and rejected [34]. In Lebanon, the names of several patients were retrieved and spread to the public for sociopolitical propagandas. As the first case of COVID-19 in the country was diagnosed coming from Iran, the area and the community associated with the patient were stigmatized. As more new cases were diagnosed, other patients belonging to different sects and residential zones emerged, and diversion of the stigma and scrutiny took place.

## Conclusions

The true burden of the COVID-19 pandemic on Lebanon should be assessed from several perspectives. The disease has affected the whole country and not only those infected or exposed. The ability to contain the infection and overcome the critical period needs a multidimensional response. It should take into consideration the health as well as the socioeconomic, educational, constitutional and various other factors. The capability of a country or a nation to overcome the epidemic relies on its preparedness, strategic reserves and the people′s trust in the government. The COVID-19 epidemic could not have come at a worse time for Lebanon. The country is on the brink of bankruptcy, the healthcare system is struggling for survival and the government is striving to regain the trust of its population.
